# Adiponectin Mediates Running-Restored Hippocampal Neurogenesis in Streptozotocin-Induced Type 1 Diabetes in Mice

**DOI:** 10.3389/fnins.2018.00679

**Published:** 2018-10-02

**Authors:** Suk-Yu Yau, Thomas Ho-Yin Lee, Ang Li, Aimin Xu, Kwok-Fai So

**Affiliations:** ^1^Department of Rehabilitation Sciences, The Hong Kong Polytechnic University, Hung Hom, Hong Kong; ^2^Guangdong-Hong Kong-Macau Institute of CNS Regeneration, Jinan University, Guangzhou, China; ^3^Department of Medicine, The University of Hong Kong, Pokfulam, Hong Kong; ^4^Department of Pharmacology and Pharmacy, The University of Hong Kong, Pokfulam, Hong Kong; ^5^The State Key Laboratory of Pharmacology, The University of Hong Kong, Pokfulam, Hong Kong; ^6^State Key Laboratory of Brain and Cognitive Sciences, Pokfulam, Hong Kong; ^7^Department of Ophthalmology, The University of Hong Kong, Pokfulam, Hong Kong

**Keywords:** hippocampal neurogenesis, physical exercise, adiponectin, streptozotocin, type I Diabetes

## Abstract

Streptozotocin (STZ)-induced diabetes impairs learning and memory performance and reduces adult hippocampal neurogenesis. Physical exercise brings beneficial effects. We have previously shown that adiponectin, an adipocyte-secreted hormone critically involved in the pathology of diabetes, is a key mediator for exercise-enhanced adult hippocampal neurogenesis. Here, we tested whether adiponectin is required for exercise to restore adult hippocampal neurogenesis in an animal model of diabetes. The findings showed that a single injection of 195 mg/kg STZ-induced diabetes significantly increased serum levels of corticosterone and reduced hippocampal adiponectin levels in adult mice. STZ injection also significantly reduced the number of Ki67 and doublecortin (DCX) positive cells and the ratio of co-labeling of DCX and bromodeoxyuridine (BrdU) in the hippocampal dentate region, indicating a decrease in adult hippocampal neurogenesis. Two-week voluntary wheel running significantly restored hippocampal neurogenesis in the diabetic wild-type mice, but not adiponectin knockout mice, indicating that adiponectin is critical for physical exercise to restore hippocampal adult neurogenesis in mice with diabetes. The results suggest that increasing adiponectin levels could be a therapeutic approach to restore hippocampal neurogenesis impairment in individuals with diabetes.

## Background

Diabetes has multifactorial causes. Genetic factor is known to play an important role, as do adopting a high-calorie diet and a sedentary lifestyle (Kharroubi and Darwish, 2015). Diabetes contributes to the development of metabolic complications and peripheral neuropathy (Kharroubi and Darwish, 2015). Moreover, epidemiological studies have demonstrated that both type 1 diabetes (insulin-deficient, T1DM) and type 2 diabetes (insulin-resistant, T2DM) are co-morbid with cognitive impairment, including dementia and Alzheimer’s disease (AD) (Umegaki, 2010; Jacobson et al., 2011; McCrimmon et al., 2012; Viscogliosi et al., 2012; Cato et al., 2014). Of note, AD is now termed “type 3 diabetes,” emphasizing the concurrence of metabolic and cognitive deficits in individuals with AD (de la Monte, 2014).

Adiponectin, an adipocyte-secreted protein hormone, acts as a regulator of glucose and fatty acid metabolism through adiponectin receptor I and receptor II (AdipoR1 and AdipoR2). A decreased adiponectin level is associated with both T2DM and AD dementia (Okamoto et al., 2006; Teixeira et al., 2013; Garcia-Casares et al., 2016). Two adiponectin receptors are widely expressed in various brain regions: AdipoR1 is highly expressed in the hippocampus, the amygdala, and the medial prefrontal cortex, whereas AdipoR2 is primarily expressed in the hippocampus, and the hypothalamus (Liu et al., 2012).

Neuroanatomical studies have shown that diabetic and AD’s dementia-like diseased rodent models share similar neuropathy in the hippocampus, including suppressed adult neurogenesis in the hippocampal dentate gyrus (DG) (Zhang et al., 2007; Ghosal et al., 2010) and dendritic atrophy and cognitive impairment (Mufson et al., 2015; Kandimalla et al., 2018). We have demonstrated that the trimeric adiponectin can cross the blood-brain barrier and up-regulate hippocampal cell proliferation via the AdipoR1-phosphorylated AMP-activated protein kinase (pAMPK)-signaling cascade (Yau et al., 2014).

Given that adiponectin is a multifunctional hormone engaged in the endocrine system and the central nervous system, recent research focuses on unveiling whether adiponectin can be a potential therapeutic target, specifically, for addressing cognitive deficits. Low adiponectin levels detected in depressed patients can be increased with antidepressant treatment (Machado-Vieira et al., 2017). Consistent with these findings, the adiponectin haploinsufficient mouse model is more susceptible to stress-induced depressive disorders, and intracerebroventricular injection of adiponectin or recombinant adenovirus-expressing adiponectin has been shown to restore neuroplasticity and depression-like behaviors in both adiponectin haploinsufficient and deficient mouse models (Yau et al., 2011; Liu et al., 2012). These findings support the potential pivotal role of adiponectin in depression-like behaviors in rodents. Changes of depression-like behavior in these rodent models could possibly be linked to impaired hippocampal dendritic and spine plasticity (Zhang et al., 2016) and reduced cell proliferation, differentiation, and survival of new-born cells in the hippocampal DG (Yau et al., 2014).

Physical exercise has strong support as an effective non-pharmacological intervention for treating diabetes (Sato et al., 2007; Hillman et al., 2008). We have previously shown that adiponectin serves as a key mediator for the beneficial effects of physical exercise on both hippocampal neurogenesis and depression (Yau et al., 2014). Accumulated studies have further demonstrated the significance of adiponectin in diabetic neuropathy (Anderson et al., 2014; Chen et al., 2015; Ji et al., 2015); however, the role of adiponectin in ameliorating diabetes-impaired neuroplasticity through physical exercise has yet to examine. Here, we sought to determine if physical exercise is capable of counteracting diabetes-suppressed adult hippocampal neurogenesis and whether this beneficial effect is through an adiponectin-dependent manner. We hypothesized that adiponectin is a key mediator for physical running to restore impairment in adult hippocampal neurogenesis in mice with diabetes.

## Materials and Methods

### Animals and Experimental Timeline

All experimental procedures were approved and followed the guidelines of the Committee on the Use of Live Animals in Teaching and Research, the University of Hong Kong. 8- to 10-week-old male wild-type (WT) C57BL/6J mice or adiponectin knock-out mice (*Adipo^-/-^*) with the same genetic background were housed with a 12-h:12-h light-dark cycle at the animal holding room at the University of Hong Kong. The animals were fed with standard chow and water *ad libitum*.

To induce type 1 diabetes mellitus, a single dose of 195 mg/kg streptozotocin (STZ) (Sigma, St Louis, MO, United States) in 0.1 M citrate buffer was delivered through intraperitoneal injection. Control mice were injected with citrate buffer vehicle only. Seven days after STZ injection, blood glucose from tail blood was measured using Accu-Chek© Performa glucometer. Mice with blood glucose levels higher than 20 mmol/dL were considered to be diabetic. Body weights and blood glucose levels were monitored weekly. Mice were group housed with the shared running wheels (one wheel for two mice) (Yau et al., 2014). This served to avoid the additional stress that was induced by social isolation. For studying hippocampal neurogenesis, proliferating cells were labeled by daily i.p., injection of BrdU (50 μg/g body weight, dissolved in 0.9% saline at a concentration of 10 mg/mL; Sigma-Aldrich, United States), during the last three days of the two weeks running period.

**FIGURE 1:**
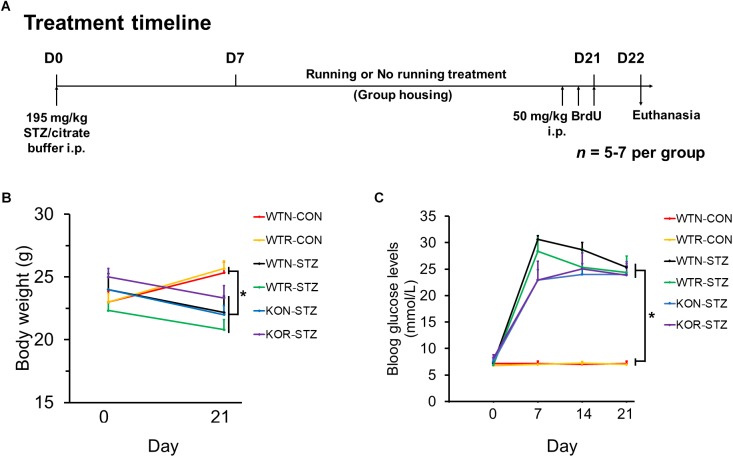
Streptozotocin significantly impeded body weight gain and increased blood glucose levels in both non-runners and runners. **(A)** Treatment timeline and animal groupings are abbreviated as follows: WTN, control wild-type non-runners; KON, control *Adipo^-/-^* non-runners; WTR, control wild-type runners; KOR, control *Adipo^-/-^* runners; WTN-STZ, diabetic wild-type non-runners; KON-STZ, diabetic *Adipo^-/-^* non-runners; WTR-STZ, diabetic wild-type runners; KOR-STZ, diabetic *Adipo^-/-^* runners. **(B)** Control mice showed body weight gain, while diabetic mice showed decreases in body weight after STZ administration. **(C)** Blood glucose levels were significantly increased by STZ administration in both WT and KO mice when compared to the untreated control mice. ^∗^*P* < 0.005 compared to WTN and WTR, respectively.

Animal grouping and the treatment timeline are illustrated in **Figure 1A**. WT and *Adipo^-/-^* mice were randomly divided into the diabetic and non-diabetic group, and then sub-divided into running or non-running group.

### Tissue Preparation

Mice were deeply anesthetized with a mixture of ketamine and xylazine. After the collection of trunk blood, they were perfused with 0.9% saline for 5 min, and 4% paraformaldehyde (PFA) in 0.01 M phosphate buffered saline (PBS) for 15 min. The isolated brains were post-fixed in 4% PFA overnight at 4°C. The brains were then transferred to 30% sucrose until they sank. The left hemisphere (1-in-6 series, 40 μm thickness), were cryosectioned using a sliding freezing microtome (Thermo Fisher Scientific, Pittsburg, CA, United States). The slices were stored in the cryoprotectant at - 20°C until use.

### Immunohistochemistry and Immunofluorescent Staining

The brain sections were retrieved in citrate buffer (pH 6.0) at 95°C for 30 min, then incubated in 2 N HCl for 30 min at 37°C and 0.1 M borate buffer (pH 8.5) for 15 min. After washing in 0.01 M PBS, the sections were incubated overnight with the anti-BrdU (1:1000, Abcam, United Kingdom), and then incubated with the biotinylated goat anti-rat IgG (1:200, Dako, Denmark). The BrdU staining was visualized with the peroxidase method (ABC system, Vector Laboratories, Burlingame, CA, United States) and diaminobenzidine kits (DAB kits, Sigma-Aldrich, United States). For doublecortin (DCX) or Ki67 staining, sections were incubated with rabbit anti-DCX (1:200; Abcam, United Kingdom) or rabbit anti-Ki67 (1:1000; Novocastra) antibody, followed by the biotinylated goat anti-rabbit (1:200; Dako, Denmark) and visualized using the same methods mentioned above.

Immunofluorescent colabeling of BrdU and DCX was performed. After antigen retrieval, sections were incubated with primary antibodies overnight and secondary antibodies for 2 h, including goat anti-rabbit IgG Alexa Fluor-488 and goat anti-rat IgG Alexa Fluor-568 (1:200, Dako, Denmark). The mounted sections were observed by fluorescent microscopy (Axioplan, Zeiss, Oberkochen, Germany).

### Quantification of BrdU ^+^, Ki67 ^+^, and DCX ^+^ Cells

BrdU ^+^, Ki67 ^+^, and DCX ^+^ cells were counted in the 1-in-6 series of six sections (from bregma - 1.34 to - 3.80 mm), using optical fractionators system (grid size: 55 μm × 55 μm; counting frame: 35 μm × 35 μm) of Stereo Investigator (MicroBrightfield Inc., Williston, VT, United States). Cells residing in the subgranular zone and granular cell layer of the DG were counted whereas those located in the uppermost focal plane were excluded. The resulting numbers were then multiplied by six to obtain the total number of cell count from the left hippocampus. Quantification was performed in the sample blinded manner.

### Quantification of DCX/BrdU Co-labeled Cells

Thirty BrdU ^+^ cells from six slices were randomly selected for calculating the co-labeling ratio with DCX, as an indicator for neuronal differentiation. Quantification was performed in a blind manner. Co-labeling was determined when a cell with a BrdU ^+^ nucleus stained together with a DCX ^+^ surrounding soma (**Figure 4D**). DCX/BrdU ratios were then computed.

### Protein Extraction

Total proteins were extracted from freshly isolated hippocampi. Tissues were lysed with radioimmunoprecipitation assay (RIPA) buffer (Cell Signaling Technology, Danvers, MA, United States), supplemented with the protease and phosphatase inhibitor cocktails, and phenylmethanesulfonyl fluoride (Sigma-Aldrich, United States). Samples were homogenized and subsequently sonicated (Branson Sonicator #2510, Branson Ultrasonics, Danbury, CT, United States), for 20 s with a 50% pulse and cleared by centrifugation (10,000 × *g*) at 4°C for 30 min. Supernatant protein was collected and stored at - 80°C until use. Supernatant protein concentrations were determined by the BCA Protein assay (Bio-Rad Laboratories, Hercules, CA, United States).

### Measurement of Serum Corticosterone, Hippocampal Adiponectin and Brain-Derived Neutrophic Factor (BDNF) Levels

Trunk blood was collected and serum samples were used for measuring corticosterone levels. Blood samples were placed at room temperature for 30 min, followed by centrifugation (Eppendorf centrifuge 5417R, Hauppauge, NY, United States) at 1,000 × *g* for 20 min at 4°C. Serum aliquots were then stored at - 80°C until use. The proteins were extracted from whole hippocampal tissues. Fresh tissues were collected 24 h after the 14-day running, and total proteins were extracted. The levels of corticosterone adiponectin and BDNF were determined using commercially available ELISA kits, including mouse CORT ELISA kits (Assay Designs, Enzo Life Sciences, Switzerland), mouse adiponectin ELISA kits (Adipogen Corporation, United States) and mouse BDNF ELISA kits (R & D system, United States) for detection in hippocampal tissues.

### Statistical Analysis

One-way repeated measure ANOVA was used to evaluate the changes in blood glucose levels following STZ injection while student *t*-test was performed to compare the levels of neurotrophic factors in STZ-induced diabetic condition and control. To demonstrate the effects of voluntary wheel running and diabetic condition on hippocampal neurogenesis in WT and adiponectin knock-out mice, two-way ANOVA was used to compare data from cell quantification, followed by Fisher’s *post hoc* test as appropriate with the SPSS 25.0 software (SPSS Inc., Chicago, IL, United States). A probability (*P*) value of less than 0.05 is considered as statistically significant. Data were shown as means ± SEM.

## Results

### Streptozotocin Injection Decreases Body Weight Gain and Induces Hyperglycaemia

First, we examined whether STZ could induce the pathophysiology resembling the clinical features of T1DM in WT and *Adipo*^-/-^ mice. Our results showed that STZ significantly (*P* < 0.005) impeded body weight gain in both WT and *Adipo*^-/-^ non-runners and runners when compared to the control WT non-runners and runners, respectively (see **Figure 1B**). In addition, drastic elevations of blood glucose levels were observed seven days after STZ administration, and the elevations were maintained over 3 weeks (see **Figure 1C**). A one-way repeated measures ANOVA comparing STZ injected mice when compared to control WT non-runners and runners, respectively, indicated successful diabetic induction in the mice (*F*(1,5) = 33.56, *P* < 0.005).

### Streptozotocin Injection Increases Levels of Corticosterone and Reduces Levels of Adiponectin

**FIGURE 2:**
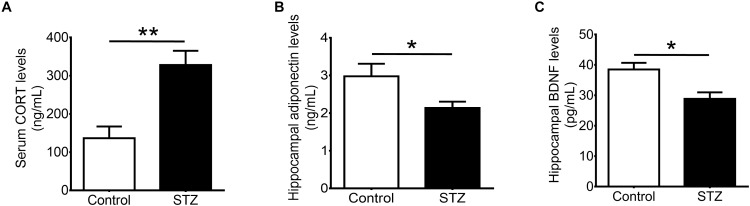
Effects of streptozotocin on corticosterone and neurotrophic factors. Intraperitoneal administration of streptozotocin significantly increased **(A)** serum levels of corticosterone while significantly reduced **(B)** hippocampal levels of adiponectin and **(C)** hippocampal levels of brain-derived neurotrophic factor in wild-type non-runners. Student *t*-test ^∗^*P* < 0.05, ^∗∗^*P* < 0.005.

Our results showed that STZ increased serum levels of corticosterone (**Figure 2A**; *t*(1,8) = 3.972, *P* < 0.005), and reduced hippocampal levels of adiponectin (**Figure 2B**; *t*(1,10) = 2.254, *P* < 0.05). Furthermore, STZ reduced hippocampal levels of BDNF (**Figure 2C**; *t*(1,7) = 3.131, *P* < 0.05).

### Voluntary Wheel Running Restores Hippocampal Cell Proliferation in STZ-Induced Diabetic Mice

**FIGURE 3:**
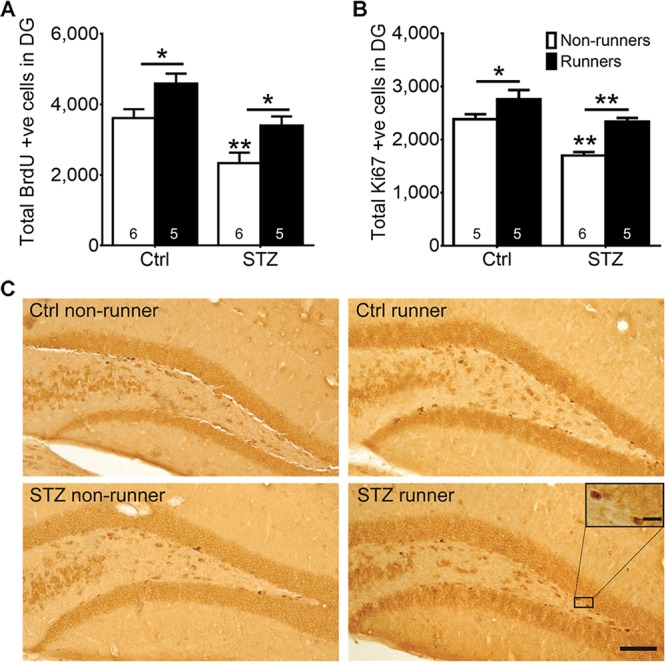
Running restored hippocampal cell proliferation in diabetic mice. STZ administration significantly suppressed hippocampal cell proliferation. **(A)** BrdU ^+^ cells in the DG, ^∗^*P* < 0.05 vs. STZ runners, ^∗^*P* < 0.05 vs. control runners, ^∗∗^*P* < 0.005 vs. STZ non-runners. **(B)** Ki67 ^+^ cells in the DG, ^∗∗^*P* < 0.005 vs. STZ runners, ^∗∗^*P* < 0.005 vs. control runners, ^∗∗^ *P* < 0.005 vs. STZ non-runners, and **(C)** Representative images of Ki67 ^+^ cells in the hippocampal DG (Scale bars, 100 μm in 100×, 10 μm in 400×).

Results of the two-way ANOVA analysis testing the hypothesis that running restores diabetes-impaired hippocampal neurogenesis revealed significant main effects for running condition and STZ on cell proliferation in the DG (**Figure 3A**; BrdU: main effect for STZ: *F*_3,22_ = 20.06, *P* < 0.0005; main effect for running: *F*_3,22_ = 13.83, *P* < 0.005; **Figures 3B,C**; Ki67: main effect for STZ: *F*_3,20_ = 30.40, *P* < 0.0001; main effect for running: *F*_3,20_ = 25.41, *P* = 0.0001). *Post hoc* analysis indicated that STZ-induced diabetes significantly reduced the number of both BrdU ^+^ (**Figure 3A**; *P* < 0.005) and Ki67 ^+^ (**Figures 3B,C**; *P* = 0.0001), cells when compared to the control non-runners, indicating that diabetic condition significantly suppressed hippocampal cell proliferation. In contrast, voluntary wheel running increased cell proliferation in control mice (**Figure 3A**; BrdU: *P* < 0.05; **Figures 3B,C**; Ki67: *P* < 0.05 vs. control non-runners), and restored the number of BrdU ^+^ and Ki67 ^+^ cells in diabetic mice (**Figure 3A**; BrdU: *P* < 0.05; **Figures 3B,C**; Ki67: *P* < 0.0005 vs. STZ non-runners).

### Voluntary Wheel Running Restores Diabetes-Suppressed Neuronal Differentiation in Wild-Type Mice

**FIGURE 4:**
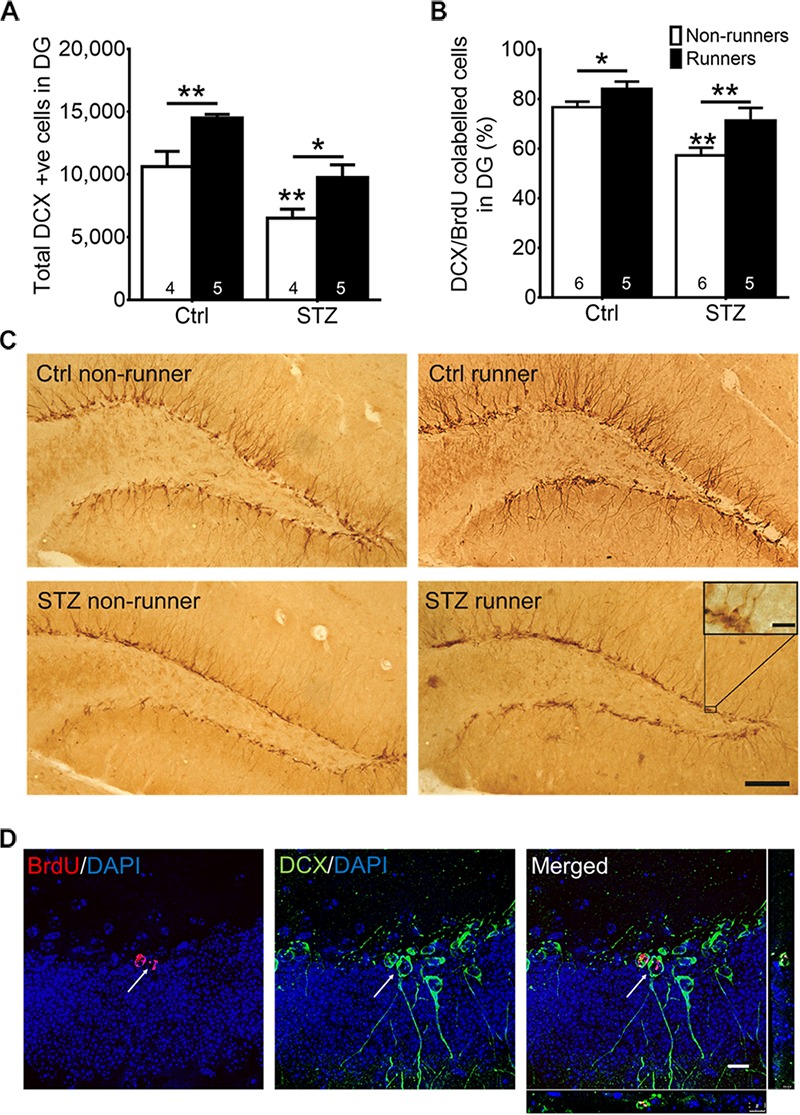
Running restored the number of newborn neurons and neuronal differentiation in the hippocampal dentate gyrus in diabetic mice. **(A)** STZ administration significantly suppressed number of immature neurons in hippocampal DG, ^∗^*P* < 0.05 vs. STZ runners, ^∗∗^*P* < 0.005 vs. control runners, ^∗∗^*P* < 0.005 vs. STZ non-runners. **(B)** Running significantly increased neuronal differentiation estimated by the co-ratio of BrdU and DCX labeling in both control, and STZ mice. ^∗∗^*P* < 0.005 vs. STZ runners, ^∗^*P* < 0.05 vs. control runners, ^∗∗^*P* < 0.005 vs. STZ non-runners. **(C)** Representative images of DCX^+^ cells cells in the hippocampal DG (Scale bars, 100 μm in 100 ×, 10 μm in 400 × ). **(D)** Representative images of BrdU/DCX co-labeling.

The two-way ANOVA testing the hypothesis that running enhances neuronal differentiation in diabetic mice revealed significant main effects for running condition and STZ injection on immature neurons in the DG (**Figures 4A,C**; effect of STZ: *F*_3,22_ = 26.64, *P* = 0.0001; effect of running: *F*_3,22_ = 17.20, *P* = 0.001). STZ-induced diabetic condition significantly decreased the number of immature neurons (**Figures 4A,C**; *P* < 0.05 vs. control non-runners). Conversely, running significantly increased the number of immature neurons in control mice (**Figures 4A,C**; *P* < 0.05 vs. control non-runners), and also restored the decrease in diabetic mice (**Figures 4A,C**; *P* < 0.05 vs. STZ non-runners). Running promoted neuronal differentiation as evidenced by an increase in the ratio of co-labeled cells (**Figures 4B,D**; *P* < 0.05 vs. control non-runners). Notably, running restored the decrease in ratio DCX/BrdU co-labeled cells in diabetic mice (**Figures 4B,D**; *P* < 0.005 vs. STZ non-runners), suggesting running could ameliorate the reduction in neuronal differentiation caused by the diabetic condition.

### Adiponectin Knockout Diminished Running-Restored Hippocampal Neurogenesis in STZ-Induced Diabetic Mice

**FIGURE 5:**
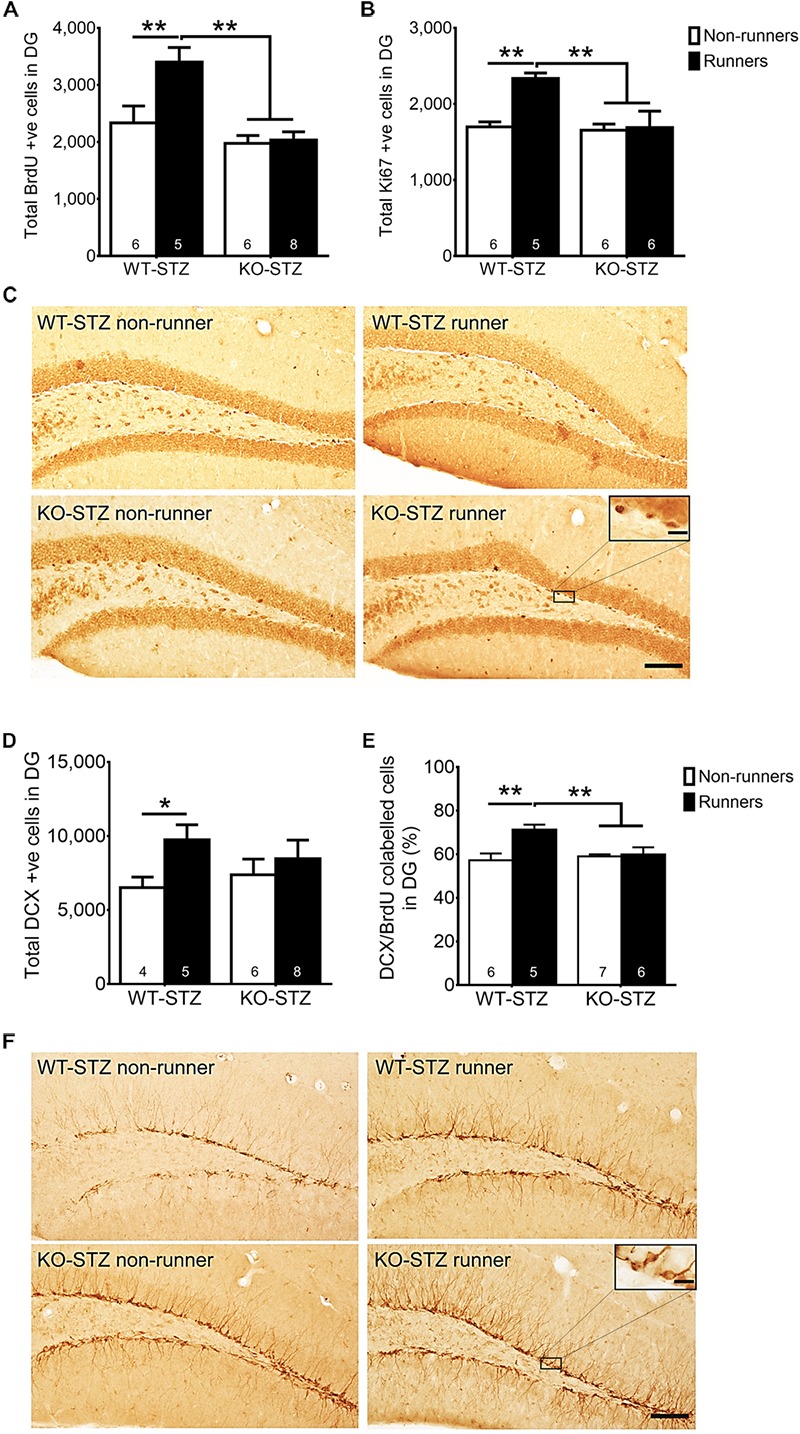
Adiponectin deficiency diminished running-restored hippocampal neurogenesis. **(A,B)** Adiponectin deficiency significantly diminished running-restored proliferating cells in diabetic mice (BrdU ^+^ cells: ^∗∗^*P* < 0.005 vs. KO diabetic runners and non-runners, respectively; Ki67 ^+^ cells: ^∗∗^*P* < 0.005 vs. KO diabetic runners and non-runners, respectively), indicating ADN is required for running-restored hippocampal cell proliferation in diabetic mice. **(C)** Representative images of Ki67 ^+^ cells in the hippocampal DG (Scale bars, 100 μm in 100×, 10 μm in 400×). **(D)** Running increased number of immature neurons in WT mice, but not *Adipo^-/-^* mice. **(E)** Running increased neuronal differentiation in wild-type diabetic mice, but not *Adipo^-/-^* mice, indicating that adiponectin is required for running-induced neuronal differentiation in diabetic mice. ^∗∗^*P* < 0.005 vs. KO diabetic runners and non-runners, respectively. **(F)** Representative images of DCX ^+^ cells in the hippocampal DG.

The two-way ANOVA testing the hypothesis that adiponectin is required for exercise to restore hippocampal neurogenesis in diabetic mice revealed main effects for genotype and running on regulating hippocampal cell proliferation (**Figure 5A**; BrdU: effect of genotype: *F*_3,22_ = 16.73, *P* = 0.0005; effect of running: *F*_3,22_ = 7.09, *P* < 0.05; **Figures 5B,C**; Ki67: effect of genotype: *F*_3,20_ = 7.06, *P* < 0.05; effect of running: *F*_3,20_ = 6.67, *P* < 0.05), and significant interactions between genotype and running (**Figure 5A**; BrdU: *F*_3,22_ = 5.70, *P* = 0.05; **Figures 5B,C**; Ki67: *F*_3,20_ = 5.37, *P* < 0.05). *Post hoc* tests showed that adiponectin knock-out diminished voluntary running-restored cell proliferation in diabetic mice (**Figure 5A**; BrdU: *P* < 0.0005 vs. STZ-KO-runners; **Figures 5B,C**; Ki67: *P* < 0.005 vs. STZ-KO-runners). Furthermore, voluntary running enhanced number of immature neurons in WT diabetic mice, but not *Adipo^-/-^* mice (**Figures 5D,F**; *P* < 0.05). Voluntary running also enhanced neuronal differentiation (**Figure 5E**; DCX/BrdU: effect of genotype: *F*_3,20_ = 6.83, *P* = 0.074; effect of running: *F_3_*_,20_ = 3.57, *P* < 0.05) as evidenced by an increased the ratio of co-labeled cells in WT diabetic mice (**Figure 5E**; *P* < 0.05); however, this effect was diminished by adiponectin knockout, indicating the indispensable role of adiponectin for running-promoted neuronal differentiation of new-born neurons in the hippocampal DG of diabetic mice.

## Discussion

The study findings showed that a high-dose STZ administration significantly decreased levels of adiponectin in the hippocampus and significantly suppressed cell proliferation and neuronal differentiation of newborn cells in the hippocampus of diabetic mice. Two-week voluntary running restored the observed impairment in hippocampal neurogenesis in the samples. However, voluntary running failed to restore hippocampal neurogenesis in diabetic mice when adiponectin was absent, indicating the indispensable role of adiponectin in exercise-promoted hippocampal neurogenesis in mice with diabetes. In short, the study findings show the critical role of adiponectin in mediating exercise-promoted hippocampal neurogenesis in mice with diabetes.

The underlying mechanisms of impaired cognitive function observed in late-stage diabetic human patients are not clear and controversial. In the present study, we attempted to mimic diabetes by impairing the peripheral endocrine system which induced neuropathy in the hippocampus, a region that governs learning and memory. It has been reported that decreased adiponectin levels and hyper-activation of the hypothalamic-pituitary-adrenal (HPA) axis are concomitant in both T2DM and depressed patients (Roy et al., 1990; Leo et al., 2006; Narita et al., 2006; Yilmaz, 2008). A possible reason for these findings could be that diabetes over-activates the HPA axis, resulting in an elevated blood corticosteroid level of diabetic animals (Chan et al., 2001), which then suppresses cell proliferation and differentiation in the hippocampal DG (Revsin et al., 2009). With the STZ injection, we observed an increase in serum corticosterone levels. Based on these findings, it would be reasonable to speculate that STZ-induced cytotoxicity in the pancreatic β-cells (Lenzen, 2008) leads to insulin-deficiency, which then causes hyperglycaemia, a characteristic symptom of diabetes. In mice, STZ-induced hyperglycaemia might have induced a hypersensitization of adrenocorticotropic hormone in the adrenal glands, which then resulted in adrenocortical growth and a subsequent increase of adrenal secretion of corticosterone to the bloodstream (Revsin et al., 2008).

High-fat diet-induced (HFD) T2D rodent models have impaired hippocampal neurogenesis and cognitive functions (Lindqvist et al., 2006), which are associated with an increased serum corticosterone levels. Spontaneous and STZ-induced T1D rodent models have been shown to have a significant reduction in cell proliferation and differentiation in the DG compared to their control littermates (Revsin et al., 2009). Similarly, T2D models exhibit a decrease in brain cell proliferation and differentiation in the DG, as shown in *db/db* mice and in HFD mice and rats (Lindqvist et al., 2006; Hwang et al., 2008; Yi et al., 2009; Boitard et al., 2012). Furthermore, STZ models of mice and rats, HFD rats, and *db/db* mice display a consistent decrease in dendritic density and branching (Ho et al., 2013). It is well-noted that the potentiation of synaptic plasticity is impaired and long-term depression can be induced in STZ-induced rat (Kamal et al., 1999, 2000). In addition, decreased hippocampal neurogenesis after STZ-induced T1D is accompanied by deficits in behavioral tasks involving spatial learning and memory (Revsin et al., 2009). *db/db* and HFD mice have also been shown to have spatial working memory deficits (Boitard et al., 2012). Taken together, STZ-induced diabetes suppresses hippocampal neurogenesis, possibly linked to elevated corticosterone levels and reduced adiponectin levels.

The beneficial effect of physical exercise on cognitive function are well-known. Several cellular and molecular processes have been suggested to explain these beneficial effects, including upregulated expression of neurotrophins, increased adult hippocampal neurogenesis, and enhanced potentiation of synaptic plasticity (van Praag et al., 1999), all three of which might be promoted by physical exercise. In the present study, our results have shown that 2-week voluntary wheel running can restore impeded adult hippocampal neurogenesis upon STZ induction, suggesting that exercise can counteract the diabetes-induced neurogenesis impairment in the hippocampus.

Diabetes is often co-morbid with cognitive impairment, for example, depression and AD. Our previous research has documented the efficacy of adiponectin in exercise-induced hippocampal neurogenesis as well as its role as a mediator in hippocampal neurogenesis together with reduction in depression-like behavior (Yau et al., 2014). In addition, *in vitro* research has shown that trimeric adiponectin promotes cell proliferation through AdipoR1-APPL1-pAMPK cascade in the adult mouse hippocampal DG-isolated neural progenitors and in Neuro2a neuroblastoma (Yau et al., 2014) and recombinant globular adiponectin stimulates p38 mitogen-activated protein kinase (p38MAPK)-glycogen synthase kinase 3 β (GSK-3 β)- β -catenin signaling cascade in the adult rat hippocampus-isolated neural progenitors (Zhang et al., 2011). In the present study, our results not only demonstrated the same beneficial effect of physical exercise and the critical role of adiponectin in a mouse model, but indirectly showed that the reduced adiponectin levels in serum (Szkudelska et al., 2014) and the hippocampus are still critical as mediators of exercise-promoted hippocampal neurogenesis in a diabetic mouse model. Recently, the role of adiponectin in the hippocampal neurogenesis has also be documented as it exhibited neurotrophic effects (Zhang et al., 2016) and improving both depression-like behaviors (Liu et al., 2012; Yau et al., 2014) and cognitive functions (Zhang et al., 2017) in mice models. Adiponectin deficiency reduces dendritic length, branching, and spine density of the dentate granule neurons, while a daily intracerebroventricular infusion of adiponectin for one week increased of dendritic spinogenesis and complexity in late-born granule neurons (Zhang et al., 2016). Moreover, fear-associated learning is impaired in adiponectin-deficient mice and is restored by intra-DG infusion of adiponectin (Zhang et al., 2017).

The study has two limitations that should be acknowledged and be cautious when interpreting the results. High-dose STZ administration is commonly used to induce T1DM in rodent models, but we are aware that there are some differences between T1DM in humans and STZ-induced diabetic rodent models. Clinical studies have shown that T1DM patients have an increased serum adiponectin level (Leth et al., 2008; Pereira et al., 2012), while the serum and hippocampal adiponectin levels were shown to decrease upon STZ administration in mice (Szkudelska et al., 2014). Future study employing *ob*/*ob* genetic model or multiple low-dose STZ with high-fructose diet-induced T1DM model should be considered to better mimic clinical T1DM. Although the STZ-induced diabetic mice model is not an exact manifestation of T1DM in human, we are able to demonstrate that physical exercise poses a strong and beneficial effect on hippocampal neuroplasticity to counteract the high toxicity of STZ and the severity of this chemically induced diabetic model, and on the other hand, to illustrate the necessity of adiponectin in mediating the exercise-promoted hippocampal neurogenesis.

BDNF and IGF-1 are essential in neuronal differentiation and survival (Rossi et al., 2006; Zhang et al., 2014), and both T1D and T2D models have been reported to have reduced BDNF levels (Nakagawa et al., 2000; Stranahan et al., 2009). In fact, we have shown STZ injection decreased hippocampal BDNF levels (data not shown). Based on this investigation, we illustrated that adiponectin is necessary to facilitate exercise-mediated hippocampal neurogenesis; hence, future study on the changes at the molecular level, such as serum and hippocampal adiponectin levels, hippocampal BDNF and IGF-1 levels, hippocampal mRNA expressions of AdipoR1 and R2, by comparing the effects of physical exercise and STZ-induced diabetics in WT and adiponectin knockout mice will be useful to connect the adipocyte-brain axis. In addition, further study on neuronal microstructures, for example, dendritic branching and spine density, can give us a new insight on the critical roles of adiponectin in hippocampal neurogenesis.

Despite these limitations, the study provides important new findings with respect to the novel role of adiponectin in mediating physical exercise-induced hippocampal adult neurogenesis in diabetic brains. To the best of our knowledge, this is the first time that the involvement of adiponectin in exercise-induced hippocampal neurogenesis in diabetic animals has been documented. Our results demonstrate that two-week wheel running promotes cell proliferation and neuronal differentiation in the hippocampus in an adiponectin-dependent manner in a diabetic mouse model. The findings further confirm the critical role of adiponectin in exercise-promoted hippocampal neurogenesis. The findings also suggest the possibility that adiponectin could be a therapeutic target for restoring degenerative neuropathy in the hippocampus in individuals with diabetes; and thus, a potential treatment for ameliorating cognitive impairment associated with diabetes. Further exploration of how exercise can modulate hippocampal neurogenesis-associated pathways interfered by diabetic conditions and how exercise could illustrate pro-cognitive effects through animal behavioral tests are warranted.

## Author Contributions

S-YY, K-FS, and AX conceived and designed the experiments and contributed reagents, materials, and analysis tools. S-YY performed the experiments. S-YY, AL, and TL analyzed the data. S-YY and TL wrote the paper.

## Conflict of Interest Statement

The authors declare that the research was conducted in the absence of any commercial or financial relationships that could be construed as a potential conflict of interest.
